# Dispersal variability and associated population-level consequences in tree-killing bark beetles

**DOI:** 10.1186/s40462-016-0074-9

**Published:** 2016-04-15

**Authors:** Markus Kautz, Muhammad Ali Imron, Kai Dworschak, Reinhard Schopf

**Affiliations:** Department of Ecology and Ecosystem Management, Technische Universität München, Freising, 85354 Germany; Present address: Institute of Meteorology and Climate Research – Atmospheric Environmental Research (IMK-IFU), Karlsruhe Institute of Technology (KIT), Garmisch-Partenkirchen, 82467 Germany; Department of Forest Resource Conservation, Gadjah Mada University, Yogyakarta, 55281 Indonesia

**Keywords:** Dispersal mortality, European spruce bark beetle, Foraging, Host selection, Individual-based modelling, IPS-model, Movement ecology, Pioneering, Population plasticity, Risk spreading

## Abstract

**Background:**

Dispersal is a key process in the response of insect populations to rapidly changing environmental conditions. Variability among individuals, regarding the timing of dispersal initiation and travelled distance from source, is assumed to contribute to increased population success through risk spreading. However, experiments are often limited in studying complex dispersal interactions over space and time. By applying a local-scaled individual-based simulation model we studied dispersal and emerging infestation patterns in a host − bark beetle system (*Picea abies* – *Ips typgraphus*). More specifically, we (*i*) investigated the effect of individual variability in beetle physiology (flight capacity) and environmental heterogeneity (host susceptibility level) on population-level dispersal success, and (*ii*) elucidated patterns of spatial and/or temporal variability in individual dispersal success, host selectivity, and the resulting beetle density within colonized hosts in differently susceptible environments.

**Results:**

Individual variability in flight capacity of bark beetles causes predominantly positive effects on population-level dispersal success, yet these effects are strongly environment-dependent: Variability is most beneficial in purely resistant habitats, while positive effects are less pronounced in purely susceptible habitats, and largely absent in habitats where host susceptibility is spatially scattered. Despite success rates being highest in purely susceptible habitats, scattered host susceptibility appeared most suitable for dispersing bark beetle populations as it ensures population spread without drastically reducing success rates. At the individual level, dispersal success generally decreases with distance to source and is lowest in early flight cohorts, while host selectivity increased and colonization density decreased with increasing distance across all environments.

**Conclusions:**

Our modelling approach is demonstrated to be a powerful tool for studying movement ecology in bark beetles. Dispersal variability largely contributes to risk spreading among individuals, and facilitates the response of populations to changing environmental conditions. Higher mortality risk suffered by a small part of the dispersing population (long-distance dispersers, pioneers) is likely paid off by reduced deferred costs resulting in fitness benefits for subsequent generations. Both, dispersal variability in space and time, and environmental heterogeneity are characterized as key features which require particular emphasis when investigating dispersal and infestation patterns in tree-killing bark beetles.

**Electronic supplementary material:**

The online version of this article (doi:10.1186/s40462-016-0074-9) contains supplementary material, which is available to authorized users.

## Background

Dispersal –defined as the movement of an individual between its natal site and the potential location of reproduction– is unquestionably important for species ecology and evolution. It ultimately drives gene flow and may reduce extinction risks for populations [1]. Therefore, dispersal is one key process in the response of natural populations which are confronted with environmental heterogeneity or habitat fragmentation [2, 3], and is assumed to ensure population stability, e.g., by reducing kin competition, avoiding inbreeding, escaping from antagonists or optimizing the exploitation of available habitats [1]. However, dispersal reveals to be a risky game for the single individual: It may suffer either from direct costs (mortality, if host or mate finding fails) or deferred costs (e.g., decrease in reproductive success). Thus, the decision whether, when and where to disperse is not only crucial in life-history for the individual itself but also implies sustained consequences for population dynamics [4, 5].

Variability among intraspecific individuals, e.g., concerning physiological, functional or behavioral traits, can largely affect population dynamics, ecological communities and species´ interactions [6, 7]. Since many dispersal processes are strongly associated with such individual traits, considering intraspecific variability is of particular importance for movement ecology. For instance, recent studies revealed that individual variability in movement behavior increases the frequency of rare dispersal events like very short- or long-distance movement in relative to a more homogeneous population (=increased leptokurtosis; [8, 9]), which in turn may alter costs and benefits among dispersing individuals. Hence, despite of being widely ignored in ecological models in the past, intraspecific variability is suggested a key element in dispersal and meta-population ecology and needs to be explicitly considered in such models [10].

For eruptive insect species such as tree-killing bark beetles (Coleoptera, Curculionidae, Scolytinae) dispersal is a key driver of population dynamics [11, 12]. It is an essential life-history trait in bark beetles since host trees become unsuitable for breeding once they have been colonized by one generation. Consequentially, offspring are forced to emerge and forage for new habitats. Because bark beetles are group-attacking insects their dispersal behavior is strongly density-controlled: The higher the number of simultaneously attacking beetles the higher the chance of overcoming host tree defense mechanisms [13]. Aggregation is mediated by kairomones originating from host trees [14], and in a second step by pheromones emitted by beetles [15]. In low population densities (=endemics) bark beetles are forced to locate susceptible host trees, e.g., weakened or pre-damaged by other disturbance agents such as drought or wind. In contrast, at high population densities (=epidemics) beetles can colonize and eventually kill even healthy, well defended trees (reviewed in [16, 17]). Bark beetle outbreaks have been shown to cause substantial multi-scale impacts on global forest ecosystems, implying both ecological and economic consequences (e.g., [18, 19]). Although host − bark beetle systems have now been studied for almost a century, reliable data on spatio-temporal patterns and trade-off mechanisms concerning dispersal, host selection and colonization remain scarce. That is most likely due to methodological constraints of empirically studying these small, cryptically living insects and the inherent system complexity. Mark-release-recapture experiments using pheromone traps do not reflect the true distribution of individuals for several reasons (discussed in [20–22]). Lab-based experiments suggest strong individual plasticity regarding flight propensity and capacity [23]. However, they cannot be transferred one-to-one into natural conditions due to the artificial environment they are performed in. When analysing dispersal on the basis of infestations [24, 25], results are inevitably biased towards successful individuals and uncertainty remains about the unsuccessful portion. The few experimental studies on temporal variability in bark beetle dispersal mainly address settlement order and its implied costs and benefits (e.g., [26–29]).

Theoretical models may supplement empirical research in order to investigate individual dispersal behavior and its population-level consequences (e.g., [30, 31]). In particular, mechanistic models are appropriate tools for understanding the hidden driving factors and mechanisms which lead to observable population patterns [32]. Dispersal in bark beetles has been simulated previously with a wide range of particular research foci [33]. However, such models rarely incorporate neither individual variability, each single stage of dispersal (i.e., emigration – movement – settlement), nor dispersal-associated costs or benefits which might be associated with one or more of these stages [34]. Addressing these issues, in this study we apply an individual-based dispersal model (IPS, [33]) which considers all three above-mentioned dispersal stages and which also takes individual variability into account. The aim of our study is twofold: it is firstly driven by the hypothesis that individual dispersal variability, facilitated by physiological variability, lead to enhanced population-level dispersal success, and, secondly, targets to elucidate obscure patterns which emerge from dispersal processes in the host − bark beetle system. In particular, we focus on three key patterns: (*i*) dispersal success, i.e. the rate of individuals which successfully find and colonize a host, (*ii*) host selectivity, i.e. the behavior to select among different hosts, and (*iii*) the resulting colonization density, i.e. the number of beetles colonizing a host. Ultimately, ecological implications from the derived patterns are discussed with particular regards to risk spreading and fitness trade-offs in bark beetle populations.

## Methods

### The IPS-model

**Fig. 1:**
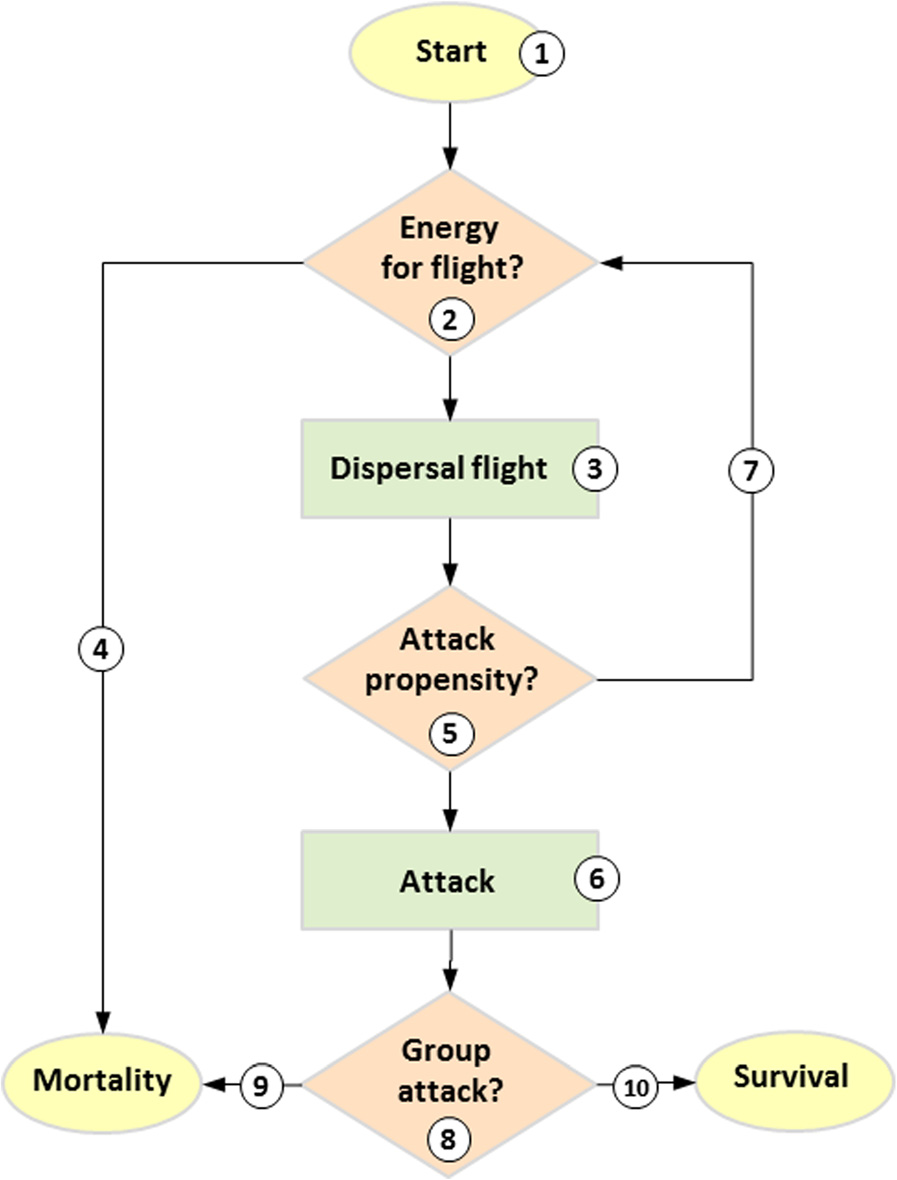
Flow chart visualizing main model processes from a beetle perspective. Each single beetle starts from a source with its individual physiology (energy level, efficiency), jointly with a specified number of conspecifics at a certain point in time (1). At every movement step the beetle checks its energetic resources (2). As long as energetic resources are sufficient it continues the dispersal flight (3), otherwise the beetle suffers mortality due to energy deficiency (4). Dispersal continuously reduces the initial energy level according to the individual-specific consumption efficiency. Movement follows a correlated random flight until the beetle perceives an attractive host within its perceptual range which pilots it directly to that host. The decision whether to attack (6) or to continue dispersal (7) is made according to the actual attack propensity (5) which is based on its fatigue level and the encountered relative host attractiveness. Once a beetle attacks a host it will depend on a certain number of conspecifics to overcome host defense mechanisms (8). Only if this threshold number is achieved the beetle attack is successful (10), otherwise the beetle suffers mortality due to host resistance (9). For details see also Table 1 and Additional file 1

The IPS-model (*Infestation Pattern Simulation*) –an individual-based, spatially explicit and local-scaled dispersal model– has been developed to facilitate insights into hidden mechanisms governing the complex host − bark beetle system. Main model concepts and processes are described below, and a more detailed model description that follows the ODD (overview, design concepts, details) protocol for describing individual-based models [35, 36] is provided in the supplementary material (Additional file 1), likewise the model version used for this study (Additional file 2). For further in-depth information regarding the model structure, validation and sensitivity analyses, we kindly refer to [33], where IPS has been previously introduced in detail. IPS was developed using the open source NetLogo environment [37], considered a standard modelling tool in individual-based ecology [38], and particularly useful for modelling movement behavior of organisms [39]. Although primarily parameterized for the most severe insect pest in European forests, the European spruce bark beetle *Ips typographus* and its host *Picea abies*, it is principally transferrable to similar systems, e.g., North American *Dendroctonus* bark beetle species. The model strictly follows a bottom-up approach, where higher-levelled system properties such as dispersal and infestation patterns emerge directly from the individual traits of its basic entities (beetles, trees) and their spatio-temporal interactions. Beetles are simulated as mobile entities capable to forage through the forest habitat, which is represented by trees covering conjoined 5x5m patches each (comprising a total forest area of ~ 500 ha). The temporal dimension is scaled in time steps, where one time step corresponds to the time a beetle needs to move from one patch to the neighboring patch; state variables are updated at every time step. Each individual, beetles and trees, is characterized by a number of parameters and traits (e.g., for beetles: energy level and consumption efficiency, movement angle, perceptual range to host cues, and for trees: susceptibility to beetles, carrying capacity, colonization status; see Table 1 for details). A model run simulates the course of a single dispersal wave, where beetles start from a centered source patch, forage through the surrounding habitat, and can aggregate and colonize an encountered host (Fig. 1, main processes are described below). A particular feature of IPS is that beetles can continuously adapt their attack propensity during the dispersal flight, according to their internal fatigue level (energetic state) and the encountered host attractiveness (due to kairomonal and/or pheromonal cues), and that host tree attractiveness responses to the varying number of attacking beetles over time. In the course of a model run the fate of each single dispersing beetle can be tracked which results either successful (infesting a host, and survival), or not (unsuccessful in host finding, mortality due to tree resistance or its own energy deficiency, which impedes continuing the dispersal). Analogously, a tree becomes either infested and suffers mortality subsequently, or it is not found by sufficient beetles and thus it remains non-infested and survives.

**Table 1:** Model input parameters for beetles and trees

	Parameter	Values	Unit	Description	References
Beetle	Energy level	10	abstract unit	Energy supplies at the start of dispersal; chosen randomly for each individual from a Gaussian distribution N (*μ*, 2), where *μ* is the used value 10; linearly reduced during flight by a constant consumption value, which is defined by the individual efficiency	[42, 43]
	Efficiency	20	abstract unit	Determines energy consumption per movement step, (consumption = 1/efficiency); chosen randomly for each individual from an exponential distribution Exp (*λ*), where 1/*λ* is the used value 20	[44]
	Perceptual range	15	meter	Radius in which an individual senses attractiveness; constant for all individuals of a dispersing population	[21, 40]
	Moving angle	45	degree	Angle of movement to neighbouring trees, related to previous movement (correlated random walk); chosen randomly in every time step for each individual from a defined sector (±45°)	[21, 41]
	Starting beetles	10,000	−	Number of beetles starting simultaneously (= flight cohort size)	-
	Total beetles	100,000	−	Total number of dispersing individuals (= source size) corresponding to a group of approx. 5 source trees	[43, 82]
	Time lag	10	time steps	Time lag between subsequent flight cohorts	-
Host	Primary attractiveness	0 − 9	abstract unit	Kairomone-induced primary attractiveness, ranging from 0 (no attractiveness) to 9 (highest attractiveness); chosen randomly from a habitat-specific range; inverse to the resistance to beetle attacks	[14, 83]
	Capacity limit	5,000	−	Maximum number of beetles infesting a host	[49]

Table modified from [33]

### Emigration and movement

Dispersal initiates randomly-directed from a simulated brood tree (=source). To account for temporal variability individual beetles start their flight in 10 subsequent flight cohorts, separated by a time lag of 10 time steps. During its simulated flight through the forest a beetle continuously verifies host tree attractiveness within its perceptual range [40] and adapts its movement and attack behavior accordingly. As long as the beetle does not identify any particular attractive host it follows a correlated random flight within a 45° right/left angle [21, 41] to the neighboring patch. In case a beetle perceives sufficiently attracting cues it will direct its flight straight toward this host. Every starting individual is equipped with an initial energy budget which is randomly chosen from a Gaussian distribution [42, 43]. Dispersal reduces this initial energy level at each movement step with an individual-specific consumption efficiency, randomly chosen from a negative exponential distribution [44]. Dispersal flight will be continued either until the individual energy level is reduced to zero, i.e., the beetle dies without finding a host, or a potential host tree is found, i.e., successful attack or death caused by tree defense.

### Host selection and attack

Decreasing energy reserves lead to increasing attack propensity [45, 46]. That means the decision, whether to initiate an attack or not, is based on the actual ratio between the energy level and the local attractiveness realized within the perceptual range. If the energy level is sufficiently low and the perceived attractiveness relatively high, the beetle will select that host to start an attack (either successful or not), otherwise dispersal will be continued. Hence, decision-making is a dynamic process because at every movement step both factors, beetles energetic state and the encountered host attractiveness, may change. Host attractiveness (or susceptibility) is primarily defined by the environmental set-up (see below), thereby reflecting the varying susceptibility (=inverse resistance) of trees within a stand. Once a host is infested by beetles its attractiveness excessively increases due to the release of aggregation pheromones by attacking beetles [47, 48] until the capacity limit is reached and attractiveness falls to zero due to repellent pheromones [48].

### Aggregation and colonization

The success of an attack depends on the density of beetles on the host. If the density fits into a range between a minimum (resistance threshold, depending on host primary susceptibility) and a maximum value (capacity limit) the beetle attack results successful. Resistance threshold ranged from 30 to 200 beetles (as a linear function of host susceptibility) and capacity limit was set constant at 5,000 beetles per tree [49]. Consequentially, colonization density, i.e., number of beetles per host, may range between the minimum and maximum value. Below the minimum value the tree is able to resist an attack with specific defense mechanisms, e.g. enhanced resin production [13]. Thus, the first attacking individuals (pioneers) are dependent on attracting sufficient conspecifics in a given time to overcome the resistance threshold; otherwise they will remain unsuccessful and die.

### Environmental heterogeneity

To account for environmental heterogeneity, i.e., different levels of host susceptibility, all simulations were carried out in three different forest habitat types: a resistant, a scattered, and a susceptible type. Every single tree was attributed a certain primary attractiveness (*PA*) which was chosen randomly for each simulation run within a pre-defined habitat-specific range (cf. [33]). Attractiveness values were abstract measures ranging from 0 (least attractive, i.e., non-host) to 9 (most attractive). The resistant habitat type consisted of trees less attractive to dispersing beetles (*PA* = 0, 1, 2, or 3). The susceptible habitat was characterized by highly attractive trees (*PA* = 6, 7, 8, or 9). The scattered habitat can be seen as semi-resistant, assumed to be most realistic in between both of those extremes, and is defined by a certain percentage *p* of highly attractive trees which are randomly scattered within a resistant habitat. By default *p* = 1 and *PA* = 8, yet different settings were used for the sensitivity analyses (Additional file 3). All three habitats were isotropic, i.e., spatially uniform in all orientations to the central source of dispersal.

### Analyses

We performed two different levels of analyses, the population level and the individual level. Firstly, we assessed the effect of within-population variability on dispersal success by modifying two beetle-related physiological input parameters, initial energy level and efficiency, which in combination determine the flight capacity (Table 1). Whereas variations in energy level are partly dependent on external factors such as host quality or colonization density, efficiency variations are assumed to be population intrinsic. In order to reveal potential effects of individual variability on population-level dispersal success, the default set-up, which includes realistic assumptions concerning both parameters (S0), was compared to two artificial scenarios with partially (S1) or completely switched-off individual variability (S2). In S1 only variability in efficiency was switched-off, i.e., set equal for all individuals to the mean value of the original distribution, whereas for energy level the default Gaussian distribution was used. In S2 any variability was completely switched-off by using the mean values of both parameters for each individual. As global output for population dispersal success the percentage of beetles which successfully infested a host was recorded. For simplicity, we omitted additional scenarios where variability is reduced in a more gradual way and focussed on these extreme cases (switched-on/off variability) instead. In order to prove robustness of the results we carried out sensitivity analyses where different settings of beetle flight capacity (energy level, efficiency) and environmental heterogeneity in scattered habitats (*p*, *PA*) were tested (Additional file 3).

Subsequently to the population-level analyses, we performed more detailed analyses on the individual level by using the most realistic default setting regarding beetles (S0) and habitats (resistant, scattered with *p* = 1 % and *PA* = 8, susceptible), in order to show patterns of spatial and/or temporal variability in individual dispersal success, host selectivity and the resulting colonization density.

Simulations were repeated 30 times for each scenario and habitat type, and model output was stored after an entire simulation run had finished. In total we simulated dispersal of almost 1 billion beetles foraging through 2 billion trees.

## Results

### Population-level patterns

#### Effects of individual variability on dispersal success

**Fig. 2:**
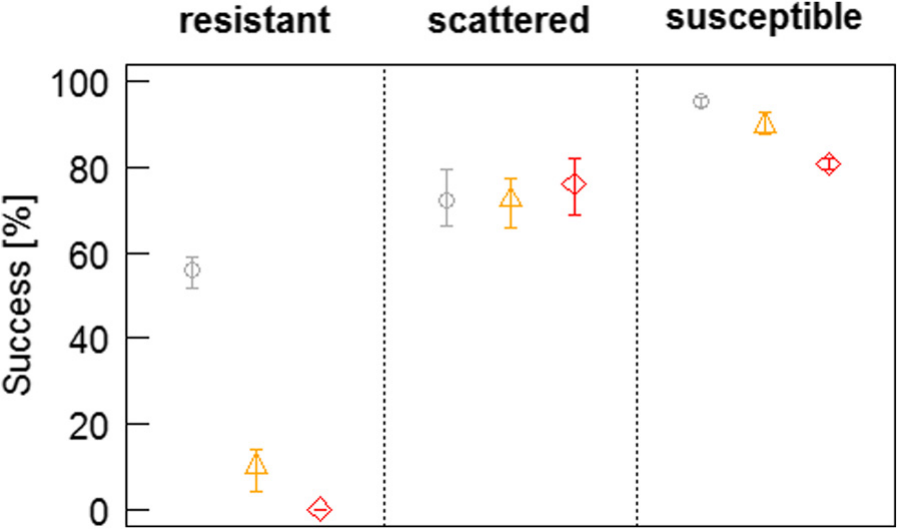
Effect of individual variability on population-level dispersal success. Compared are success rates in three different variability scenarios: full variability (S0, grey circle), reduced variability (S1, orange triangle), and completely switched-off variability (S2, red diamonds) for each of the three habitats. Error bars show the mean and extreme values (min, max) of 30 repetitions

Individual variability in flight capacity (energy level, efficiency) significantly enhances population-level dispersal success in the resistant habitat, and lead to slightly increased success in susceptible habitats (Fig. 2, Additional file 3). In both habitats success rates are highest with highest variability (S0) and decreased consistently with decreasing variability (S1, S2). As an extreme case, completely switched-off variability (S2) lead to 100 % dispersal mortality in resistant habitats and impede any population persistence. In contrast, there is no such beneficial effect in scattered habitats; a reduction in variability has no (S1) or slightly negative effects (S2) on success rates (Fig. 2). Sensitivity analyses indicate a general influence of both flight capacity and environmental setting on the variability effect: the lower the flight capacity and the less attractive the scattered habitat, the more beneficial individual variability is for population success (Additional file 3).

### Individual-level patterns

#### Dispersal success

**Fig. 3:**
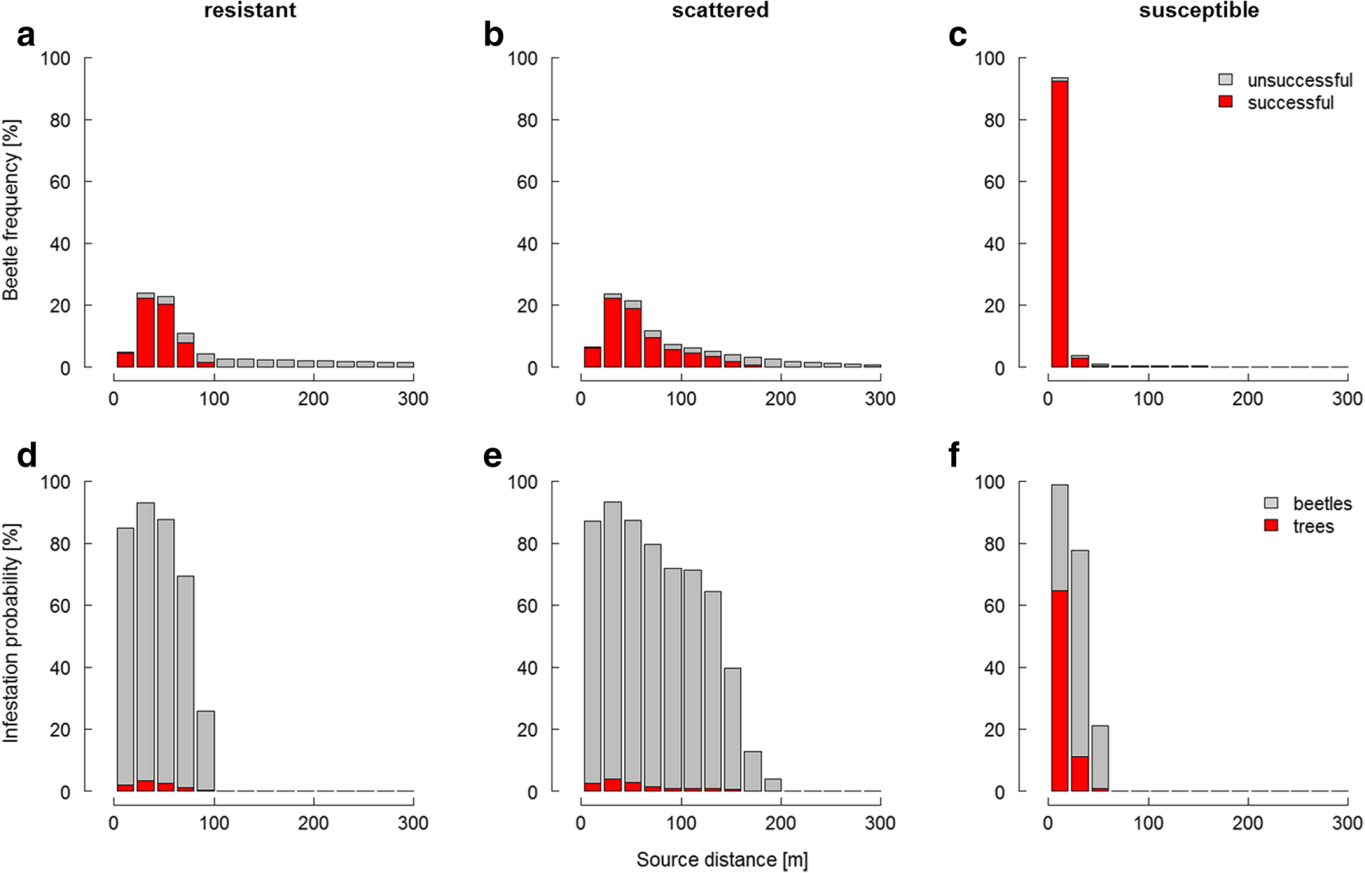
Dispersal success and infestation probability as a function of distance-to-source in different habitat types. The upper panel (**a** − **c**) shows successful and unsuccessful beetles as percentage of the total dispersing population. The lower panel (**d** − **f**) indicates the probability for a beetle to successfully infest a tree and for a tree to become infested. Columns represent 20 m-distance classes; more than 90 % of all beetles are included in the shown 300 m in each habitat type

Individual dispersal patterns are strongly distance-to-source dependent, irrespective of the simulated habitat: Increasing distance to source negatively affects beetle frequency, the ratio of successful beetles, and the probability for a tree to become infested (except in the extreme vicinity in the resistant and scattered habitats; Fig. 3). Leptokurtosis of the resulting dispersal kernel increases from scattered towards resistant habitats, and is highest in susceptible habitats (Fig. 3a − c). Long-distance dispersers are exposed to a particularly high mortality risk in resistant stands (Fig. 3a and d), while beetle success in the close vicinity to a source is greater than 80 %, regardless the habitat type. Across all three habitat types the scattered one provides the optimal combination of relatively high success and large dispersal distances, which both are beneficial for bark beetles populations (Fig. 3). For all applied settings of scattered habitats success rates are fairly high (>65 %), with spreading distances being notably larger than in homogeneous habitats (Additional file 3). Resistant habitats provide the worst environment for population persistence (low success, medium distance), and in susceptible habitats the population is only maintained locally without the potential for spreading (high success, short distance).

**Fig. 4:**
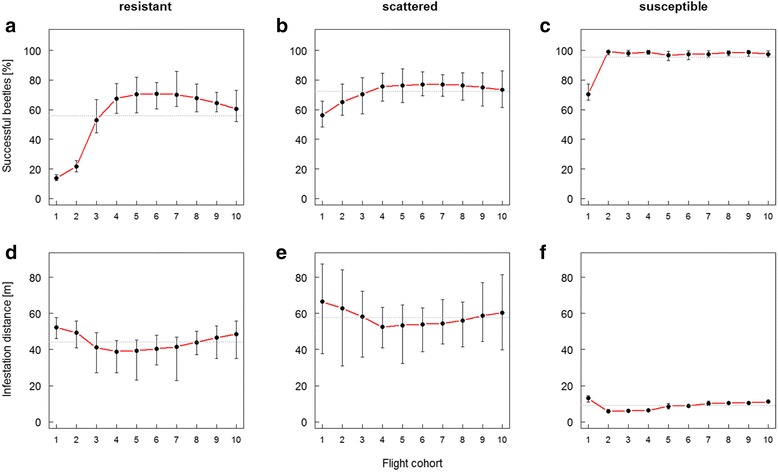
Dispersal success and distance as a function of timing in different habitat types. Shown are success rates (**a** − **c**) and infestation distances (**d** − **f**) of dispersing beetles from the 10 subsequent flight cohorts. Error bars show the mean and extreme values (min, max) of 30 repetitions. Dotted horizontal lines mark the mean values over all flight cohorts

Regarding temporal dispersal variability, success was inhomogeneously distributed among the 10 subsequent flight cohorts in all three habitat types (Fig. 4a − c). The first (pioneering) flight cohort was suffering highest mortality during dispersal. Mortality in pioneers is particularly severe in resistant habitats where first cohorts suffered mortality about three times higher than delayed dispersers (1^st^ cohort: 86 % mortality; 2^nd^: 78 %; Fig. 4a). Intermediate flight cohorts (4^th^ to 8^th^) experienced the lowest mortality rates, whereas mortality was increasing again towards the last cohorts. A similar pattern was observed in the scattered habitat (Fig. 4b), but with success rates being higher compared to the resistant one. In contrast, in the susceptible habitat only the very first cohort suffered a considerable higher mortality. Subsequent cohorts were characterized by a constantly high dispersal success (Fig. 4c).

Additionally, the spatial distribution of beetles from different flight cohorts was determined by the host distance to the source: Across all habitats, closer-to-source hosts were mainly colonized by intermediate flight cohorts, while more distant hosts are disproportionately more frequented by early and late cohorts (Fig. 4d − e).

### Host selectivity

**Fig. 5:**
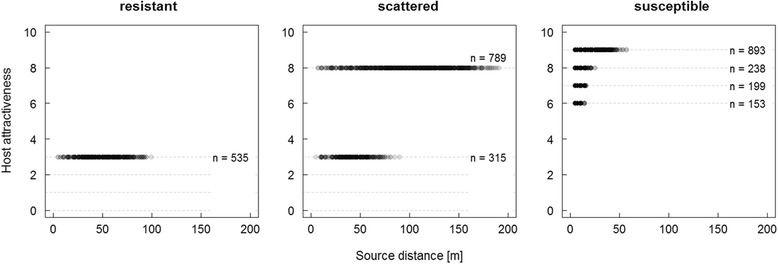
Host selectivity as a function of distance to source in different habitat types. Each grey dot represents one infested tree indicating its primary attractiveness (*n*). Dashed horizontal lines mark the range of primary attractiveness present in each habitat type

Host susceptibility level plays a crucial role determining infestation patterns: Hosts with higher primary attractiveness were more frequently colonized than less susceptible hosts, attractiveness of colonized host decreased with distance, and least susceptible hosts (with *PA* < 3) did not become infested, irrespective of their location due to the insufficient beetle density for a successful attack (Fig. 5). Consequentially, host selectivity of beetles increased with distance to source, that means the farther beetles disperse the more dependent they become to find susceptible hosts for successful attacks.

### Colonization density

**Fig. 6:**
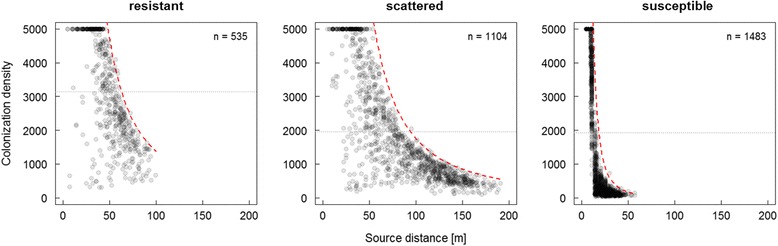
Colonization density as a function of distance to source in different habitat types. Each grey dot represents one infested tree indicating its colonization density (*n*). Dotted horizontal lines mark the mean value over all infested trees, and the dashed *f* = *ax* ^*-b*^ -shaped function visualizes the emerging strongly distance-dependent limit in number of beetles colonizing a tree

The number of successful beetles colonizing a single host decreased with increasing source distance in all three habitat types (Fig. 6). Complete colonization of hosts (colonization density = capacity limit) occurred only in source − host distances < 50 m in resistant and scattered habitats and < 10 m in the susceptible habitat. In farther distances a negative power law function clearly delimits the colonization density towards the maximum in all habitats. The mean colonization density was highest in the resistant habitat (3,141 beetles/host) compared to the scattered (1,965) and susceptible one (1,928; Fig. 6).

## Discussion

### Individual variability enhances risk spreading

The effect of individual variability in flight capacity on population success in host finding was revealed as clearly habitat-dependent (Fig. 2, Additional file 3): Reduced variability does at most minimally increase dispersal success in scattered habitats, and the advantage of a more variable population only shows when host susceptibility shifts towards extremes. In both the susceptible and resistant habitat types, dispersal success notably decreases with reduced variability, particularly eminent in resistant stands. Such environmental extremes might occur due to a variety of local-scale factors, e.g., tree physiology [13, 50], pre-damages such as windthrow or snowbreak [16], stand structure and exposition [51, 52], or regional-scale factors such as drought [53, 54]. These effects can (temporarily) transform a resistant habitat into a scattered or even into a susceptible habitat. Thus, maintaining variability enables populations to quickly react to unforeseeable, changing environments and contributes to population stability. Hence, our findings emphasize risk spreading through spatial dispersal as crucial for bark beetles which are highly affected by their stochastic environment, particularly in critical endemic population stages when host resistance is a strong limiting factor [55].

### Dispersal patterns in space and time

By providing the missing link between the spatial distribution of total dispersing individuals, and that of individuals successfully infesting a host (=dispersal kernel), our results go a step beyond of what previous studies on eruptive herbivores have achieved. For the first time, the two distributions, as well as the potential factors affecting them, can be analysed in a single system. Whereas total dispersal is shown to be distinctively long-tailed (cf. [56]), the resulting kernel of infesting individuals is of a much more leptokurtic nature (Fig. 3). This is in general accordance with separately observed distributions of dispersing individuals [20, 57] and infestations [24, 25], which are best fitted by either negative exponential or power law functions. Our results indicate a strong effect of both dispersal distance and timing on mortality. This can be explained by the group-attacking behavior in bark beetles which requires certain synchronization, i.e., density of attacking individuals, to overcome host defense. Dispersal success and spatial spread is also shown as highly sensitive to the environmental setting (cf. [58]). According to our results, the scattered habitat is suggested to be most suitable for a dispersing bark beetle population, with the optimal compromise between a relatively high success rate and distant spatial spread of individuals. From the forest management perspective, these scattered susceptible hosts, e.g., single uprooted or broken trees, should not be neglected in order to effectively reduce the risk of bark beetle disturbances. In the more homogeneous habitats, the resistant and the susceptible one, spatial spread is limited (<100 m), likely caused by several reasons. Resistant habitats offer less susceptible hosts for an attack. Thus, beetles can only be successful in very high attack densities which only occur in close vicinity to the source. Beetles in susceptible habitats mainly colonize hosts close to the source due to the surplus of susceptible trees which results in increased survival (over all flight cohorts) yet reduced spatial spread of the population (Fig. 3 and 4). Variance in beetle success and corresponding distance is lowest in susceptible habitats since beetles are hardly restricted to find susceptible hosts (Fig. 4c and f).

There is scarce empirical evidence on total dispersal success within a bark beetle population in order to validate our simulated mortality rates. For pioneers there are a few studies on *D. ponderosae* indicating mortality up to 70 % [59, 60] which are in general accordance with our model results for resistant stands. Another study revealed higher average dispersal mortality of 57 % for *D. frontalis* [61]. Previous modelling approaches simulated the complete range of mortality rates (0 − 100 %) using a set of scenarios differing in host availability and/or search strategies [62, 63]. Given the remarkable potential range in dispersal mortality and its high relevance for meta-population ecology, future efforts on its empirical quantification seem desirable.

Host selectivity in bark beetles is shown to be a function of population density, and is thus strongly distance dependent, which is in line with previous experimental and modelling studies [43, 64, 65]. Furthermore, since density also varies over time, host selectivity can be assumed to vary not only in space but also in time, suggesting that pioneers are more selective than joiners.

Interestingly, highest colonization densities occur in resistant habitats (Fig. 6). This can be explained by the higher number of simultaneously attacking beetles required for a successful colonization compared to less resistant habitats. Once this threshold is overcome, a host is particularly attractive in relation to its neighbors, and hence accumulates beetles. In all three habitats, a negative power law function marks the distance-dependent limit of colonization density (Fig. 6). That means that under the given assumption of an isotropic habitat the more distant an infested host is located in relation to the source, the less densely it is colonized. This model output is noteworthy in as much as empirical data on colonization densities regarding a spatial distance-to-source gradient is absent so far. Previous studies investigated colonization densities typically with regard to site or tree conditions and subsequent reproductive success (e.g., [66]). Knowledge on colonization density related to dispersal distances, i.e. on the dispersal kernel, may gain particular significance for population dynamics as it provides information on deferred dispersal costs and benefits potentially affecting the fitness of subsequent generations.

### Costs and benefits associated with dispersal variability

Dispersal distances are assumed to evolve directly as an evolutionary consequence of incremental costs and benefits [67, 68]. In the case of bark beetles as group-attacking species, long-distance dispersers obviously suffer from particularly high direct costs as their chance to find a host and consequentially their chance to reproduce is low. Thus, within a dispersing population there are a high number of individuals following a relatively safe strategy and just a minor proportion goes (or is forced to go) for risk. Such strongly safety-biased distribution has been revealed most favourable for population performance [69]. Nevertheless, risky dispersal is an essential part of a diversified risk-spreading strategy and not without potential benefits. The higher direct dispersal costs of long-distance dispersers, however, are weight-off by individual benefits in the rare case of a distant host finding success. Since colonization densities distinctly decrease with distance (Fig. 6), long-distance dispersers are favoured by reduced deferred costs, i.e., less brood competition resulting in higher reproductive success [42, 43], and a spatio-temporal lead over antagonists which reduce subsequent mortality [70]. Finally, spatial dispersal is known to be an effective strategy to avoid inbreeding depression within populations and enhance genetic diversification [1]. However, whereas direct costs (dispersal mortality) emerged explicitly as model output, deferred dispersal costs (affecting subsequent generations) can be deduced but not explicitly shown as our simulations only cover a single dispersal wave.

Along with the strong distance dependency, timing of dispersal considerably affects the chance of a beetle to be successful in host finding and colonization. Principally, in group-attacking populations pronounced temporal dispersal variability would not be convenient because they benefit from high spatio-temporal density. A successive dispersal in bark beetles is, rather than an active behavior, mainly a consequence of external factors: (*i*) varying brood conditions cause variations in development time until an offspring can finally disperse, (*ii*) suitable weather conditions for dispersal (temperature > 16 °C, no precipitation, less wind), and (*iii*) different warming of the hibernation site in spring (tree or soil) trigger temporal dispersal frequencies. Nevertheless, our study clearly demonstrates that temporal variability affects direct costs among individuals (Fig. 4). Early starting cohorts suffer from particularly high mortality as a large part of them are pioneers being exposed to host defenses. Pioneering in bark beetles is assumed to be a desperation strategy to attack when internal energy reserves are too low to continue the flight rather than an active decision or an inherited predisposition for pioneering [21, 27]. Consequentially, pioneers prefer less vigorous host trees [28]. Our simulations show that pioneering is most important in resistant habitats where it is difficult to overcome host defenses (Fig. 4). Except for the susceptible habitat, intermediate flight cohorts are surprisingly more successful in attacking hosts than late dispersers. This is partly explained by the highest beetle densities occurring in these intermediate cohorts (cf. [26]) but also due to heterogeneous distance distributions over time (Fig. 4d − f). Obviously, early cohorts (mainly pioneers) are forced to fly farther, most likely up to their energetic limit until desperately attacking a non-colonized host tree. Delayed flight cohorts (joiners) may easily encounter trees under attack and join them until the maximum capacity is reached. For intermediate cohorts it is thus not necessary to fly farther away, as long as there are sufficient attacked (now highly attractive) hosts available close to the source. Late cohorts suffer from the complete occupation of closer hosts which now turned to be least attractive. Hence, they are forced to disperse farther and suffer an increased mortality risk. Consequentially, leptokurtosis in dispersal and infestation gradients may vary over time, a fact which seems to be barely considered in modelling studies where usually a simplistic mean kernel is assumed for a dispersing population.

However, when pioneers are successful they would be favoured by a threefold advantage of being first: Firstly, they may choose most suitable breeding sites along the stem, thereby optimizing the brood´s nutritional conditions which may likely lead to increased offspring fitness [29]. Secondly, their brood has a temporal advance over delayed colonizers (joiners). This could be crucial to ensure the brood to be in a mature stage before hibernation. Non-mature development stages (eggs, larvae) have been shown to have mortality rates of up to 100 % during hibernation ([71] and references therein). Finally, the predation risk is likely to increase with time [27, 72, 73].

Our findings demonstrate that deferred costs of dispersal may play a considerable role –in addition to direct costs– and should not be neglected in distance-based trade-off analyses. Moreover, our results support the hypothesis that bark beetle population dynamics are strongly governed by intrinsic density-dependent trade-offs [33, 74, 75].

### Advances in movement ecology of bark beetles

Studying movement ecology in small, obscured living organisms, such as tree-killing bark beetles, is at least difficult, if not impossible unless modelling approaches are used. The application of IPS, a comprehensive individual-based model, enables for the first time quantifying emerging dispersal patterns, and investigating its causes, mechanisms and consequences at the individual- and population-level in bark beetles. Because of the high economic and ecological impacts bark beetles can have on global forests, modelling of such species as disturbance agents gained increasing attention in recent years [76]. In particular, population dynamics and the resulting spatial distribution of infestations have been simulated at the stand- to landscape scale [75, 77–80]. Our study explicitly (*i*) focus on dispersal patterns at the local scale, (*ii*) comprise all basic components determining movement, i.e., internal state, navigation and motion capacity, and external factors [81], (*iii*) consider individual variability in physiology and associated behavior, e.g., flight capacity, departure time, movement angle, and attack propensity, and (*iv*) account for intra- and interspecific communication during dispersal, all of which have not, or only partly, been considered by previous modelling studies. We thus consider our detailed mechanistic approach a novel and useful contribution –in addition to lab- and field-based approaches– to study movement ecology in host − bark beetle systems.

## Conclusions

By means of an individual-based model we revealed previously obscured dispersal patterns in a host − bark beetle system, suggesting dispersal variability as efficient risk spreading strategy. Both spatial and temporal dispersal variability causes higher mortality in a small part of the population (long-distance dispersers, pioneers), whereas the majority of individuals follow a safer, more conservative strategy (short-distance dispersers, joiners). To some degree deferred benefits payoff high direct dispersal costs of risky dispersal. Intermediate individuals, i.e. medium-distance, medium-time dispersers, may optimally trade-off incremental costs and benefits, being thereby most favoured in a certain range of mean environmental conditions (cf. [67]). In contrast, the safety and the risky sub-populations account for a more diversified risk-spreading strategy which is advantageous in response to sudden changes into more extreme environmental conditions. Thus, we assume that a combination of both spatial and temporal dispersal enhances resilience and ensures population persistence in environments which are highly heterogeneous in space and time. Consequentially, our results, though limited in time scale, emphasize dispersal variability as a significant contribution to long-term population stability within bark beetles.

## Additional files

Additional file 1: Detailed model description using the ODD protocol. (DOCX 42 kb) Additional file 2: Model version used for this study (IPS 1.1). (NLOGO 31 kb) Additional file 3: Sensitivity analyses applying different settings of flight capacity and environmental heterogeneity. (DOCX 4948 kb)
