# Supplementary material for: Dispersal variability and associated population-level consequences in tree-killing bark beetles
Source: Mov Ecol. 2016 Apr 15;4:9. doi: 10.1186/s40462-016-0074-9 (PMC4832482; doi:10.1186/s40462-016-0074-9)
Supplement: Additional file 1: — Detailed model description using the ODD protocol. (DOCX 42 kb) [file 40462_2016_74_MOESM1_ESM.docx]

**Additional file 1:**

**Detailed model description using the ODD protocol**

The description of the model follows the standardized ODD-protocol (overview, design concepts, details [35, 36]) in order to facilitate better comprehension and comparability with regard to other models.

**Overview**

***Purpose***

The main purpose of the model is to understand the emerging dispersal and infestation patterns in a host−bark beetle system, which are affected by varying individual parameters in both beetle and tree-related traits. It follows a rigorous bottom-up approach, in which system traits emerge solely from the individual traits of the entities (beetles, trees) and their interactions.

***Entities, state variables, and scales***

There are two types of individuals in IPS: bark beetles are represented by mobile entities (turtles), and trees are given by 5x5 m patches. Beetles can be distinguished by a number of individual traits determining flight and host selection behavior (e.g. energy level, moving angle, attack propensity), whereas their dispersal environment, i.e. every potential host tree within a stand is characterized by its susceptibility and colonization status. The entire simulation environment comprises 501x501 patches (=single trees), i.e. a quadratic-shaped forest stand covering 627.5 ha. However, for the analyses only the inner circle of the quadrat is relevant (r = 1250 m; A = 490.9 ha). The source where the beetles start to disperse is represented by a single patch centered in the stand. A model run simulates the course of a single dispersal wave, which in nature usually takes no longer than a few days [59], and which is finished when all beetles have completed their dispersal flight either successfully (=surviving, i.e. infesting) or unsuccessfully (=dead, not infesting). The temporal dimension is scaled in time steps, where one time step corresponds e.g. to the time a beetle needs to move from one patch to the neighboring patch. An entire simulation run may include up to approx. 1500 time steps depending on the scenario simulated.

***Process overview and scheduling***

Main processes simulated include dispersal, host selection, aggregation and host colonization (see *Submodels* below). In the course of a model run every beetle suffers one of the following fates: (*i*) it successfully finds and infests a host, and survives, or (*ii*) it does not find any susceptible host and dies due to the tree´s resistance or its own energy deficiency, which impedes continuing the dispersal. Analogously, a tree (*i*) gets either infested and dies, or (*ii*) it is not found by the minimum number of beetles and thus it remains non-infested, i.e. it survives. The agent processing sequence sets the beetles first, followed by the trees. State variables of both agents are updated at every time step.

**Design concepts**

***Emergence***

System-level traits such as dispersal and infestation patterns, including the derived mortality rates for the beetle and the tree, emerge solely from individual traits and interactions of the two system´s entities.

***Adaptation***

Adaptive behavior is represented implicitly here and involves both beetles and trees. The energy level of dispersing beetles decreases by a constant consumption value per time step. In turn, their propensity to attack increases. The individual decision about whether or not to attack depends not only on its own energy level but also on the availability of attractive hosts. Both factors may vary during the beetle´s dispersal flight.

Tree´s attractiveness is initially defined by its primary attractiveness, but may increase subsequently due to attacking beetles which release pheromones. Below a minimum value (resistance threshold) this increase in attractiveness can be assumed as moderate. Exceedance of this value means that the tree´s resistance is completely overcome, resulting in intensified pheromone production which ultimately leads to a stronger increase in (secondary) attractiveness [47, 48]. On reaching the maximum capacity the tree´s attractiveness falls to zero [49]. Attractiveness can be interpreted as inverse resistance: The more resistant a tree, the less attractive it is for beetles to attack.

***Objectives***

Beetles aim to successfully find a new host tree for colonization on the one hand, and to achieve an optimized spatial spread to forage new habitats on the other hand. As measurements evaluating both aspects the model output includes the success rate (=inverse mortality rate) within the dispersing beetle population and the maximum infestation distance to the source. In the same way potential host trees strive to survive an attack by defending themselves. IPS evaluates their number, infestation rate and spatial location.

***Sensing***

Beetles are able to sense their energy level, movement direction, the attraction of trees located within the defined perceptual range (=sensing radius), and finally the number of aggregated beetles on an encountered host tree. Thus, sensing strongly affects their dispersal and infestation behavior (see *Submodels*). Trees sense the number of beetles attacking and change their status respectively from not infested to infested. Its attractiveness (=inverse resistance) also depends directly on the attack density.

***Interaction***

Interactions among model entities include attraction and rejection mechanisms affecting host selection decisions (see *Submodels*). In natural host−bark beetle systems these interactions are triggered by inter- and intraspecific volatiles: kairomones (tree–beetle) and pheromones (beetle–beetle). While kairomones play an important role in a pioneer attack, indicating a suitable host tree to the beetle by its primary attractiveness [14, 83], attractive pheromones enhance the aggregation of beetles later on and minimize the risk of unsuccessful attacks. Finally, repellent pheromones prevent any attack above a critical tree capacity threshold [49]. Interaction space is defined by the perceptual range [40].

***Stochasticity***

Randomized processes are involved in several model steps: (*i*) the determination of the individual energy level from a Gaussian-like population distribution N (µ, 2); (*ii*) the determination of individual consumption efficiency from a negative exponential distribution Exp (λ); (*iii*) the CRW-movement where the movement angle is chosen from a direction sector (-45° to 45°); and (*iv*) the primary attractiveness of trees is chosen from a pre-defined range and spatially distributed in the stand by random choice.

***Observation***

Although simulations can be observed continuously over the course (i.e. in every time step) in this study we were particularly interested in system´s response variables, which were recorded after a simulation run was completed. All observations consider the inner circle of the quadratic-shaped simulation environment covering net distances from the source of up to 1250 m.

**Details**

***Initialization***

A simulation starts with the dispersal of the beetles from the centered source. At the beginning all beetles have the status dispersing and all trees are not infested. Depending on what scenario is running different beetle- and tree-related traits are given.

***Submodels***

*Dispersal:* Dispersal initiates randomly-directed from a simulated brood tree (=source). To account for temporal variability in dispersal propensity individual beetles start their flight in subsequent flight cohorts. During its simulated flight through the forest a beetle verifies host tree attractiveness within its perceptual range [40] and adapts its movement behaviour accordingly. As long as the beetle does not identify any particular attractive host it follows a correlated random flight within a 45° right / left angle [21, 41]. In case a beetle perceives attracting cues it will direct its flight straight toward this host. Every starting individual is equipped with an initial energy budget which is randomly chosen from a Gaussian distribution [42, 43]. Dispersal reduces this initial energy level at each movement step with an individual-specific consumption efficiency (randomly chosen from a negative exponential distribution [44]). Dispersal flight will be continued until either the individual energy level is reduced to zero (i.e. the beetle dies without finding a host) or a potential host tree is found (i.e. successful attack or death caused by tree defense).

*Host selection:* Decreasing energy reserves lead to increasing attack propensity [45, 46]. That means the decision, whether to initiate an attack or not, is based on the actual ratio between the energy level and the local attractiveness. If the energy level is sufficiently low and the perceived attractiveness relatively high, the beetle will select the host to start an attack (either successful or not), otherwise dispersal will be continued. Hence, decision-making is a dynamic process because at every movement step both factors, beetle´s energetic state and the encountered host attractiveness, may change. Host attractiveness (or susceptibility) is primarily defined by the environmental set-up, thereby reflecting the varying susceptibility (=inverse resistance) of trees within a stand (effect of kairomones). Once a host is infested by beetles its attractiveness excessively increases due to the release of aggregation pheromones by attacking beetles [47, 48].

*Aggregation and colonization:* The success of an attack depends on the density of beetles on the host. If the density fits into a range between a minimum (resistance threshold, depending on host´s primary susceptibility) and a maximum value (capacity limit, constant for all hosts) the beetle attack results successful. Resistance threshold ranged from 30 to 200 beetles (cf. [33]) and capacity limit was constant at 5,000 beetles per tree [49]. Below the minimum value the tree is able to resist an attack with specific defence mechanisms, e.g. enhanced resin production [13]. Thus, the first attacking individuals (pioneers) are dependent on attracting sufficient conspecifics in a given time to overcome the resistance threshold; otherwise they will remain unsuccessful and die. If the host reaches its maximum capacity it becomes unattractive to other beetles due to repellent pheromones [48]. Consequentially, colonization density (i.e. number of beetles per host) may range between both values.
