# Supplementary material for: Dispersal variability and associated population-level consequences in tree-killing bark beetles
Source: Mov Ecol. 2016 Apr 15;4:9. doi: 10.1186/s40462-016-0074-9 (PMC4832482; doi:10.1186/s40462-016-0074-9)

**Additional file 3:**

**Sensitivity analyses applying different settings of flight capacity and environmental heterogeneity**

**Part A.** The figure displays population-level success (a) and maximum dispersal distance (b) in different habitat types, with the red dots indicating default scenarios as used for individual-level analyses. For the scattered habitat nine different settings were tested, which vary in the primary attractiveness (*PA*) and the percentage of highly susceptible hosts (*p*). For each group of *PA* scenarios (*PA* = 7, 8 and 9) the three error bars represent *p* = 0.5%, 1% and 2% from left to right. Error bars show the mean and extreme values (min, max) of 30 repetitions.

Increased attractiveness of the scattered habitat (higher values of *PA* and *p*) slightly increase success and distance. Nevertheless, the scattered habitat type consistently provide the best combination of success and distance compared to susceptible (intermediate) and resistant habitats (worst).


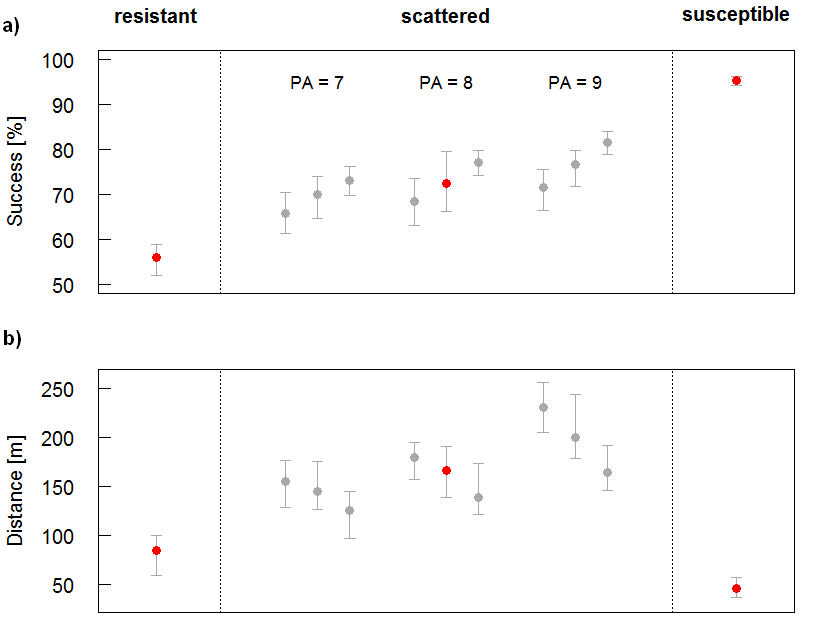


**Part B.** The figure displays the effect of individual variability in flight capacity on population-level dispersal success, i.e., the difference of success with reduced variability (S0−S1, orange triangle) and with completely switched-off variability (S0−S2, red diamond), in different habitats and with different settings of flight capacity parameters. Similar to part A of this appendix, for the scattered habitat nine different settings were tested, which vary in the primary attractiveness (*PA*) and the percentage of highly susceptible hosts (*p*). For each group of *PA* scenarios (*PA* = 7, 8 and 9) the three symbols represent *p* = 0.5%, 1% and 2% from left to right for each of the two differences. Moreover, different flight capacity settings were tested, i.e., energy level was set to 8 (a), 10 (b, default) and 12 (c), and efficiency was set to 10 (left panel), 20 (center panel, default) and 30 (right panel). Each symbol represents the mean of 30 repetitions.

Largest effects, i.e., highest differences between the S0 and the S1 or S2 scenarios, are visible in resistant habitats, irrespective of the flight capacity setting. For susceptible habitats variability effects are mainly neutral, and in few cases slightly positive, over all flight capacity settings. For scattered habitats effects can be slightly positive, neutral or slightly negative, depending on flight capacity and habitat setting. Both flight capacity and attractiveness of the scattered habitat tend to be negatively correlated with the two differences. In other words, the higher the flight capacity (towards right and lower panels), and the more attractive the scattered habitat (higher values of *PA* and *p*), the less positive (or even negative) is the effect of individual variability for population-level success.


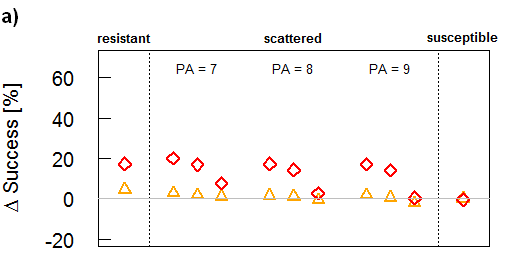

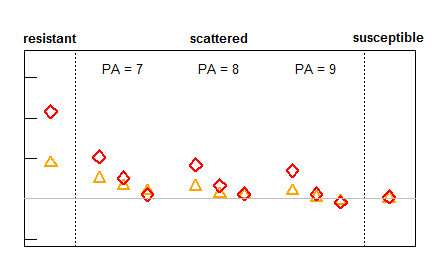

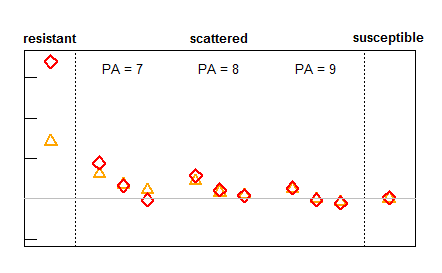


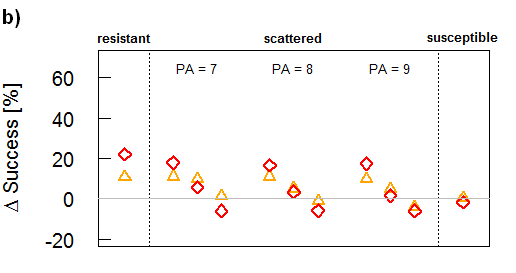

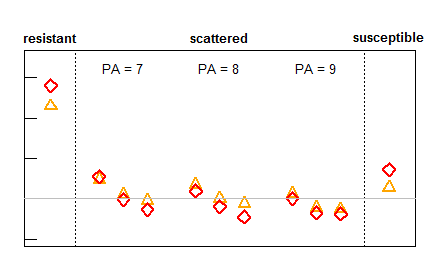

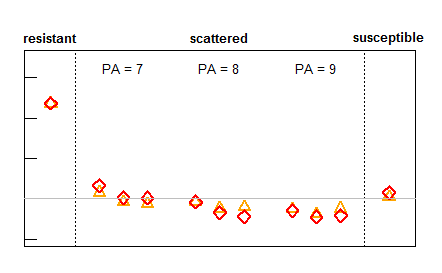


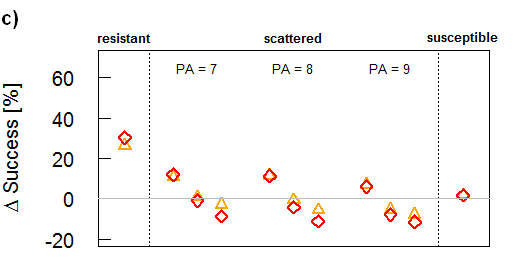

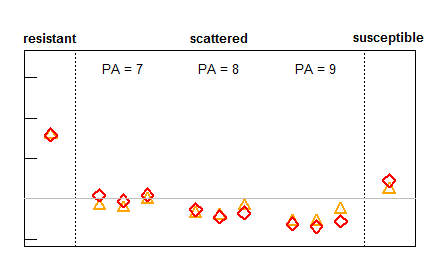

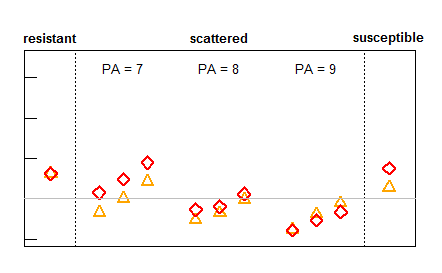

Supplement: Additional file 3: — Sensitivity analyses applying different settings of flight capacity and environmental heterogeneity. (DOCX 4948 kb) [file 40462_2016_74_MOESM3_ESM.docx]
